# Circular RNAs to predict clinical outcome after cardiac arrest

**DOI:** 10.1186/s40635-022-00470-7

**Published:** 2022-10-28

**Authors:** Francesca M. Stefanizzi, Lu Zhang, Antonio Salgado-Somoza, Josef Dankiewicz, Pascal Stammet, Christian Hassager, Matthew P. Wise, Hans Friberg, Tobias Cronberg, Alexander Hundt, Jesper Kjaergaard, Niklas Nielsen, Yvan Devaux

**Affiliations:** 1 Cardiovascular Research Unit, Department of Population Health, Luxembourg Institute of Health, 1A-B rue Edison, 1445 Strassen, Luxembourg; 2 Department of Cardiology, Clinical Sciences, Lund University and Skane University Hospital, 221 85 Lund, Sweden; 3 Department of Intensive Care Medicine, Centre Hospitalier de Luxembourg, 1210 Luxembourg, Luxembourg; 4 Department of Cardiology B, The Heart Centre, Rigshospitalet University Hospital, 2100 Copenhagen, Denmark; 5 Department of Intensive Care, University Hospital of Wales, Cardiff, CF14 4XW UK; 6 Department of Anesthesia and Intensive Care, Clinical Sciences, Lund University and Skane University Hospital, 221 85 Malmö, Sweden; 7 Department of Neurology and Rehabilitation Medicine, Clinical Sciences, Lund University and Skane University Hospital, 221 85 Lund, Sweden; 8 Integrated BioBank of Luxembourg, Luxembourg Institute of Health, Dudelange, Luxembourg; 9 Department of Anesthesia and Intensive Care, Clinical Sciences, Lund University and Helsingborg Hospital, 25187 Lund, Sweden; 10 Department of Life Sciences and Medicine, Faculty of Science, Technology and Medicine, University of Luxembourg, 4365 Esch-sur-Alzette, Luxembourg

**Keywords:** Out-of-hospital cardiac arrest, Biomarkers, Prognostication, Circular RNAs

## Abstract

**Background:**

Cardiac arrest (CA) represents the third leading cause of death worldwide. Among patients resuscitated and admitted to hospital, death and severe neurological sequelae are frequent but difficult to predict. Blood biomarkers offer clinicians the potential to improve prognostication. Previous studies suggest that circulating non-coding RNAs constitute a reservoir of novel biomarkers. Therefore, this study aims to identify circulating circular RNAs (circRNAs) associated with clinical outcome after CA.

**Results:**

Whole blood samples obtained 48 h after return of spontaneous circulation in 588 survivors from CA enrolled in the Target Temperature Management trial (TTM) were used in this study. Whole transcriptome RNA sequencing in 2 groups of 23 sex-matched patients identified 28 circRNAs associated with neurological outcome and survival. The circRNA circNFAT5 was selected for further analysis using quantitative PCR. In the TTM-trial (*n* = 542), circNFAT5 was upregulated in patients with poor outcome as compared to patients with good neurological outcome (*p* < 0.001). This increase was independent of TTM regimen and sex. The adjusted odds ratio of circNFAT5 to predict neurological outcome was 1.39 [1.07–1.83] (OR [95% confidence interval]). CircNFAT5 predicted 6-month survival with an adjusted hazard ratio of 1.31 [1.13–1.52].

**Conclusion:**

We identified circulating circRNAs associated with clinical outcome after CA, among which circNFAT5 may have potential to aid in predicting neurological outcome and survival when used in combination with established biomarkers of CA.

**Supplementary Information:**

The online version contains supplementary material available at 10.1186/s40635-022-00470-7.

## Introduction

Cardiac arrest (CA) remains one of the major public health burdens worldwide, causing up to 20% of deaths in Europe [1]. Furthermore, among comatose patients admitted to the intensive care unit on average only 40–50% survive to hospital discharge [2]. Therefore, being able to predict the outcome of these patients would aid in delivering personalized medicine, focusing on prioritization of resources and informing relatives at an earlier stage.

Current guidelines recommend the use of multiple prognostic approaches that combine neurophysiological tests, neuroimaging and biomarker assessment to predict the outcome of patients after CA [3–5]. The majority of the current biomarkers are brain-enriched markers released in the bloodstream after disruption of the blood–brain barrier, such as neuron-specific enolase (NSE) [6–8], neurofilament light chain (Nfl) [9] and S100 [10, 11]. Although the predictive value of these biomarkers has been reported in several studies [10–12] they do not seem to be accurate enough, as some of them may reflect other clinical disorders independent of CA [13–15]. Therefore, the identification of new biomarkers with additional prognostic power to be used in combination with the already established predictive modalities could improve the prognostication of CA patients.

Given the ubiquitous roles revealed in both physiological and pathological conditions, RNAs are emerging as promising biomarker candidates [16, 17]. Particularly a class of non-coding RNAs (ncRNAs), called circular RNAs (circRNAs), seem to represent the optimal intrinsic characteristics to function as biomarkers [18]. CircRNAs present a closed loop-ended structure, which originates from backsplicing [19]. This structure makes this class of RNA particularly stable when compared to any other class of linear RNAs, as they are resistant to exonuclease degradation [20]. Typically, circRNAs are more than 200 nucleotides long and lack a 5′-terminal cap and 3′-terminal poly A tail [19–21]. They also show a high cell type and tissue specificity and are abundantly expressed in numerous evolutionarily conserved human genes [22]. In addition, circRNAs are widely distributed in body fluids, where biomarkers can easily be detected. The study of this class of RNAs is still in its infancy; however, it is becoming clear that they are relevant during disease initiation and progression [23, 24] and present some potential as disease biomarkers [25–27]. Furthermore, the stability, specificity, and abundant expression in body fluids of circRNAs make them particularly advantageous as biomarkers to be assessed by minimally invasive and low-cost methodologies such as quantitative PCR (qPCR) [28].

The current work represents a substudy of the Target Temperature Management after out-of-hospital cardiac arrest trial, whose purpose was to evaluate the beneficial effect of two different targeted temperature regimens on the outcome of patients after OHCA, 33 °C versus 36 °C [29, 30]. Here, we report the potential biomarker value of a circulating circRNA, hsa_circ_0006845 (named herein circNFAT5), in OHCA prognostication.

## Methods

See Additional file 3 for additional methods.

### Patients

The TTM-trial enrolled 950 unconscious adults admitted at the intensive care unit after OHCA of presumed cardiac cause. The recruitment occurred between November 11, 2010 and January 10, 2013 in 10 countries. The trial compared the effects of two targeted temperature regimens (33 °C and 36 °C) on survival until the end of the trial and 6-month neurological outcome. Neurological outcome was assessed with the Cerebral Performance Category (CPC) score and the modified Rankin Scale (mRS). A good neurological outcome was defined as patients with none or mild-to-moderate neurological damage (CPC1-2 or mRS 0–3), while a poor outcome was defined as patients with severe neurological damage, comatose or dead (CPC3-5 or mRS 4–6).

Informed consent was waived and obtained from each patient or relatives in line with the declaration of Helsinki and the legislation of each of the participating countries. The TTM-trial is registered at www.clinicaltrials.gov (NCT01020916). The details of the design, protocol, statistical analyses and results of the trial have been discussed elsewhere [29, 31, 32].

Whole blood samples were collected in PAXgene™ Blood RNA tubes (PreAnalytiX, cat n. 762,165) 48 h after return of spontaneous circulation (ROSC). Following collection, the samples were stored at the Integrated Biobank of Luxembourg (IBBL) in compliance with the International Society for Biological and Environmental Repositories Best Practices. RNA extractions were performed and quantified using accredited methods (ISO 17025:2005). Each recruiting center in the trial decided independently whether or not to participate in the biobank study. Among the patients recruited, PAXgene™ Blood RNA samples 48 h after ROSC were available for 643 patients and RNA samples for 588 patients (Additional file 3: Fig. S1).

### Statistical analyses

Sigma Plot software (version 12.5) was used to perform statistical analyses. The *T*-test and Mann–Whitney *U* test were used to measure the differential expression levels of circNFAT5 according to the neurological outcome, targeted temperature regimen or sex. Chi-squared test or Fisher’s exact test were used to compare the categorical characteristics of TTM patients according to their neurological outcome (good vs. poor outcome). Mann–Whitney *U* test was used for continuous variables.

The neurological outcome of patients was assessed 6 months after OHCA. Patients were dichotomized as good or poor neurological outcome according to the CPC score and mRS score.

From 588 TTM patients used in this study, two sex-matched groups of 23 patients, one group with CPC1 and one with CPC5, were selected in the discovery phase for whole transcriptome RNA-seq. CircRNAs having differential expression profiles with *p* < 0.05 and log2-fold change > 0.5 or < − 0.5 between the good (CPC 1) and bad (CPC 5) outcome groups were selected for further validation. A logistic regression analysis assessed the association of circNFAT5 levels with 6-month neurological outcome while Kaplan–Meier survival curves and Cox proportional hazards models estimated the association between circNFAT5 and 6-month survival.

In the logistic regression analysis, patients were dichotomized in two groups, according to their CPC score and mRS score. Patients with a CPC 1–2 or mRS 0–3 were considered as having a good neurological outcome. Patients with a CPC 3–5 or mRS 4–6 belonged to the group with a poor neurological outcome. Both univariate and multivariable logistic regression analyses were performed. In multivariable analyses, the same clinical covariates used in previous publications [6, 33, 34] were considered: age, sex, first monitored rhythm, bystander cardiopulmonary resuscitation (CPR), circulatory shock on admission, targeted temperature regimen, time from CA to ROSC, initial serum lactate levels and NSE levels. Missing data were imputed using missForest R package (https://doi.org/10.1093/bioinformatics/btr597). Forest plots showing the odds ratios (OR) with 95% confidence interval (CI) were generated. The Akaike Information Criterion (AIC) and Hosmer and Lemeshow test were used to estimate the goodness of fit for the models. The lower AIC value, the better model fit. The Likelihood Ratio Test (LRT) was used to compare models. The net reclassification improvement (NRI) and the integrated discrimination improvement (IDI) were computed to evaluate the ability of circNFAT5 to reclassify patients misclassified by a clinical model. These analyses were performed using R version 4.0.3 with the following packages: ROCR, Hmisc, rms, lmtest, matrixStats and glmtoolbox.

Cox proportional hazards regression models were used in survival analyses. We calculated the Harrell’s C-index (the concordance index) to evaluate the univariate and multivariable Cox models. We estimated the goodness of fit of the Cox models with AIC and Grønnesby and Borgan test. We compared different Cox models using LRT. The survival analysis was performed using survival, survMisc and lmtest R packages. Kaplan–Meier curves were generated for circNFAT5 using the Youden index at the cut-off value of 0.55.

## Results

### Study flowchart

RNA extracts from whole blood samples collected in PAXgene RNA tubes and obtained 48 h after ROSC from a total of 588 patients from the TTM-trial were used in the present study (Additional file 3: Fig. S1). We first conducted a discovery phase using RNA-seq in two sex-matched groups of 23 patients from the TTM-trial. The first 2 groups of 23 patients with sufficient RNA for RNA-seq and qPCR validation were selected. The first group consisted of patients who survived and recovered without neurological sequelae at 6 months after OHCA (CPC 1), while the second group included patients who died (CPC 5) (Additional file 3: Fig. S1). Demographics and clinical characteristics of these 46 patients can be found in Table 1.

**Table 1:** Demographic and clinical characteristics of the discovery cohort

Characteristics	Neurological outcome		*p*-value
	CPC1 (n = 23)	CPC5 (n = 23)	
Age, years	61 (41–80)	74 (53- 90)	**0.002**
Sex			
Male	20 (87%)	19 (82.6%)	1
Female	3 (13%)	4 (17.4%)	
Co-morbidities			
Hypertension	7 (30.4%)	11 (47.8%)	0.19
Diabetes mellitus	2 (8.7%)	5 (21.7%)	1
Known IHD	3 (13%)	12 (52.2%)	0.093
Previous MI	2 (8.7%)	9 (39.1%)	0.502
Heart failure	1 (4.3%)	2 (8.7%)	1
COPD	1 (4.3%)	4 (17.4%)	0.174
Previous cerebral stroke	1 (4.3%)	3 (13%)	1
First monitored rhythm			
VF or non-perfusing VT	22 (95.7%)	18 (78.3%)	0.865
Asystole or PEA	1 (4.3%)	4 (17.4%)	
ROSC after bystander defibrillation	–	1 (4.3%)	
Witnessed arrest	20 (87%)	20 (87%)	0.356
Bystander CPR	16 (69.6%)	17 (74%)	0.318
Time from CA to ROSC, min	20 (8–45)	29 (11–65)	**0.02**
Initial serum lactate (mmol/l)	3.2 (0–17)	4.7 (0–16)	0.244
NSE 48 h after ROSC (ng/ml)	15 (6.6–49.2)	62.1 (8.8–291.2)	**< 0.001**
Shock on admission	2 (8.7%)	8 (34.8%)	0.111

Demographic and clinical characteristics of two groups of 23 TTM patients in the RNA-seq study according to neurological outcome established with CPC score. Continuous variables are indicated as median (range), while categorical characteristics are reported as number (frequency). A *p*-value < 0.05 was considered as statistically significant (in bold). *COPD* chronic obstructive pulmonary disease, *CPR* cardio-pulmonary resuscitation, *PEA* pulseless electric activity, *VF* ventricular fibrillation, *VT* ventricular tachycardia, *NSE* neuron-specific enolase

The remaining 542 TTM patients were used in a validation phase with measurement of candidate circRNA by quantitative PCR. Therefore, in the validation phase 256 patients with CPC 3–5 and 253 patients with mRS 4–6 showed a poor neurological outcome 6 months after OHCA, while 286 with CPC 1–2 and 289 with mRS 0–3 had a good outcome (Additional file 3: Fig. S1).

### Discovery phase: selection of circRNA candidates from RNA-seq data

**Fig. 1:**
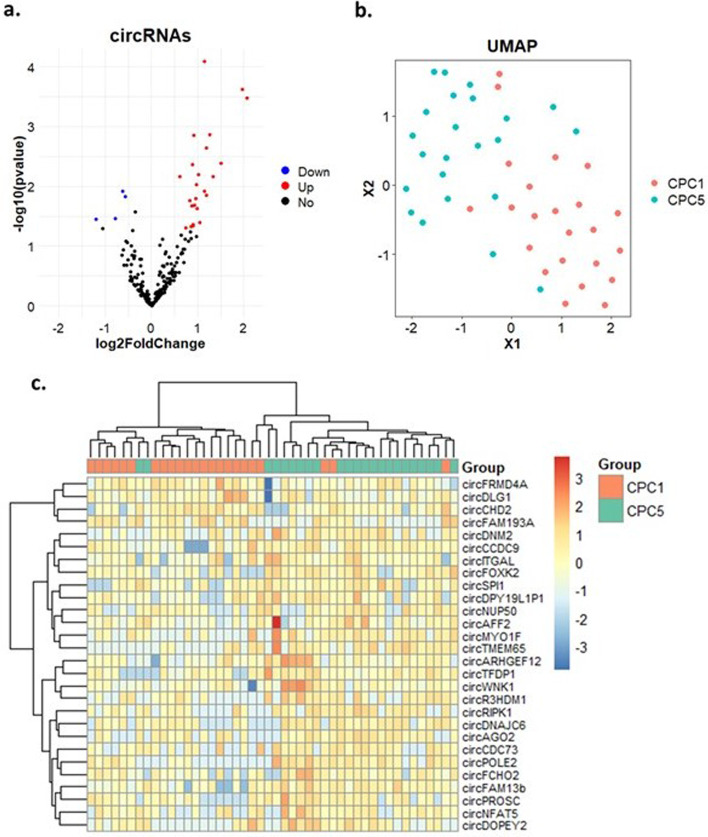
RNA-seq results from the discovery study in 46 TTM-trial patients. **a** Volcano plot showing the differential expression of the circRNAs in the CPC5 group as magnitude of change (log2-fold change) on the X-axis versus statistical significance (-log10) on the Y-axis. Color code: red, circRNAs significantly upregulated in the CPC5 group; blue, circRNAs significantly downregulated in the CPC5 group; black, circRNAs with no significant change. **b** UMAP analysis showing the clustering of the patients with X1 and X2 representing the distance between samples. Color code: red, patients with CPC1; blue, patients with CPC5. **c** Heatmap representing the expression levels of 28 circRNAs in each of the 46 RNA-seq samples. Color code: red, higher expression; blue, lower expression

Whole transcriptome RNA sequencing of the 46 patients enrolled in the discovery phase allowed the identification of 28 candidate circRNAs with differential expression profiles with *p* < 0.05 and log2-fold change > 0.5 or < − 0.5 between good (CPC 1) and poor (CPC 5) outcome groups (Table 2). Among these circRNAs, 24 were upregulated and 4 were downregulated in the CPC 5 group, as displayed by the volcano plot of Fig. 1a. These circRNAs were able to reasonably distinguish the two groups of patients as showed by the uniform manifold approximation and projection (UMAP) clustering technique (Fig. 1b). A heatmap shown in Fig. 1c displays the clusters of patients and standardized expression levels of the 28 circRNAs for each patient.

**Table 2:** List of 28 circRNAs selected from RNA-seq

Name	FPKM	Log2-fold change	*p*-value
circAFF2	28.88	0.62	0.007
circARHGEF12	9.13	1.02	0.006
circFRMD4A	8.6	− 0.57	0.015
circDLG1	8.49	− 0.62	0.012
circWNK1	5.87	0.92	0.001
circFAM13b	5.74	1.15	< 0.001
circITGAL	4.87	0.83	0.017
circTFDP1	4.15	0.98	0.009
circFCHO2	4.01	0.88	0.021
circDPY19L1P1	3.98	0.92	0.021
circCCDC9	3.72	0.88	0.004
circPROSC	3.09	0.95	0.016
circDNAJC6	2.72	0.74	0.049
circCHD2	2.63	− 0.78	0.034
circFOXK2	2.35	1.19	0.002
circNFAT5	2.17	0.99	0.024
circSPI1	2.05	1.26	0.001
circPOLE2	2.04	1.19	0.014
circRIPK1	2	1.15	0.012
circCDC73	1.95	0.89	0.044
circNUP50	1.9	0.86	0.047
circDNM2	1.77	1.34	0.007
circMYO1F	1.74	1.5	0.004
circR3HDM1	1.69	1.04	0.04
circDOPEY2	1.63	1.97	< 0.001
circAGO2	1.52	0.9	0.045
circTMEM65	1.32	2.07	< 0.001
circFAM193A	1.13	− 1.19	0.036

A positive log2-fold change indicates a higher level in poor outcome patients (CPC 5) as compared to good outcome (CPC 1) patients. Abbreviation: FPKM (Fragments Per Kilobase of transcript per Million mapped reads).

Pairs of divergent primers for qPCR were designed and tested for the 28 circRNAs listed in Table 1. Detectability of the circRNAs in blood samples (cut-off set at Ct value < 31) as well as amplification of the corrected product was confirmed for 5 circRNAs (Additional file 3: Fig. S2). Among these 5 circRNAs, circNFAT5 was selected for further analysis as it represented the best compromise between PCR primers efficiency (Additional file 3), expression levels in PAXgene samples, confirmed circularity, with 76% resistance to RNase R treatment (Additional file 3: Fig. S3) and confirmed product amplification by Sanger sequencing using divergent primers (Additional file 1 and Additional file 3: Fig. S4a) with the junction point present in the middle of the sequence (Additional file 3: Fig. S4b).

### Validation phase: assessment of the biomarker potential of circNFAT5

Circulating levels of circNFAT5 were measured by qPCR in whole blood samples collected 48 h after ROSC in the patients of the TTM-trial not enrolled in the discovery phase (*n* = 542; Additional file 3: Fig. S1). 241 patients were subjected to a targeted temperature regimen of 33 °C and 301 patients were assigned to 36 °C. 134 patients treated at 33 °C and 155 treated at 36 °C showed a good neurological outcome with CPC 1–2. Demographic and clinical characteristics of these patients are gathered in Table 3 using CPC score and Additional file 3: Table S1 using mRS score. As compared to patients in the good outcome group (CPC 1–2), patients with poor outcome (CPC 3–5) were older, had more often co-morbidities (hypertension, diabetes mellitus, heart failure and chronic obstructive pulmonary disease), had a higher delay between CA and ROSC, had higher levels of lactate, and had more often a shock on admission (Table 3).

**Table 3:** Demographic and clinical characteristics of TTM cohort using CPC score

Characteristics	Neurological outcome		*p*-value
	CPC1-2	CPC3-5	
	*n* = 286	*n* = 256	
Age, years	60 (20–90)	68 (35–94)	**< 0.001**
Sex			
Male	238 (83.2%)	205 (80.1%)	0.405
Female	48 (16.8%)	51 (19.9%)	
Co-morbidities			
Hypertension	101 (35.3%)	124 (48.4%)	**0.003**
Diabetes mellitus	31 (10.8%)	44 (17.2%)	**0.044**
Heart failure	7 (2.4%)	20 (7.8%)	**0.008**
COPD	18 (6.3%)	31 (12.1%)	**0.027**
First monitored rhythm			
VF or non-perfusing VT	260 (90.9%)	170 (66.4%)	**< 0.001**
Asystole or PEA	17 (5.9%)	77 (30.1%)	
ROSC after bystander defibrillation	7 (2.4%)	1 (0.4%)	
Unknown	2 (0.7%)	8 (3.1%)	
Witnessed arrest	262 (91.6%)	223 (87.1%)	0.118
Bystander CPR	229 (80.1%)	168 (65.6%)	**< 0.001**
Time from CA to ROSC, min	20 (0–160)	30 (0–170)	**< 0.001**
Initial serum lactate (mmol/l)	4.4 (0–20)	6.5 (0–21.3)	**< 0.001**
NSE 48 h after ROSC (ng/ml)	15 (2.5–119.1)	63.3 (3.1–782)	**< 0.001**
Shock on admission	23 (8%)	38 (14.8%)	**0.01**

Demographic and clinical characteristics of 542 patients of the TTM-trial according to neurological outcome established with CPC score. Continuous variables are indicated as median (range), while categorical characteristics are reported as number (frequency). Statistically significant *p*-values (< 0.05) are highlighted in bold in the table. Abbreviations as in Table 1

**Fig. 2:**
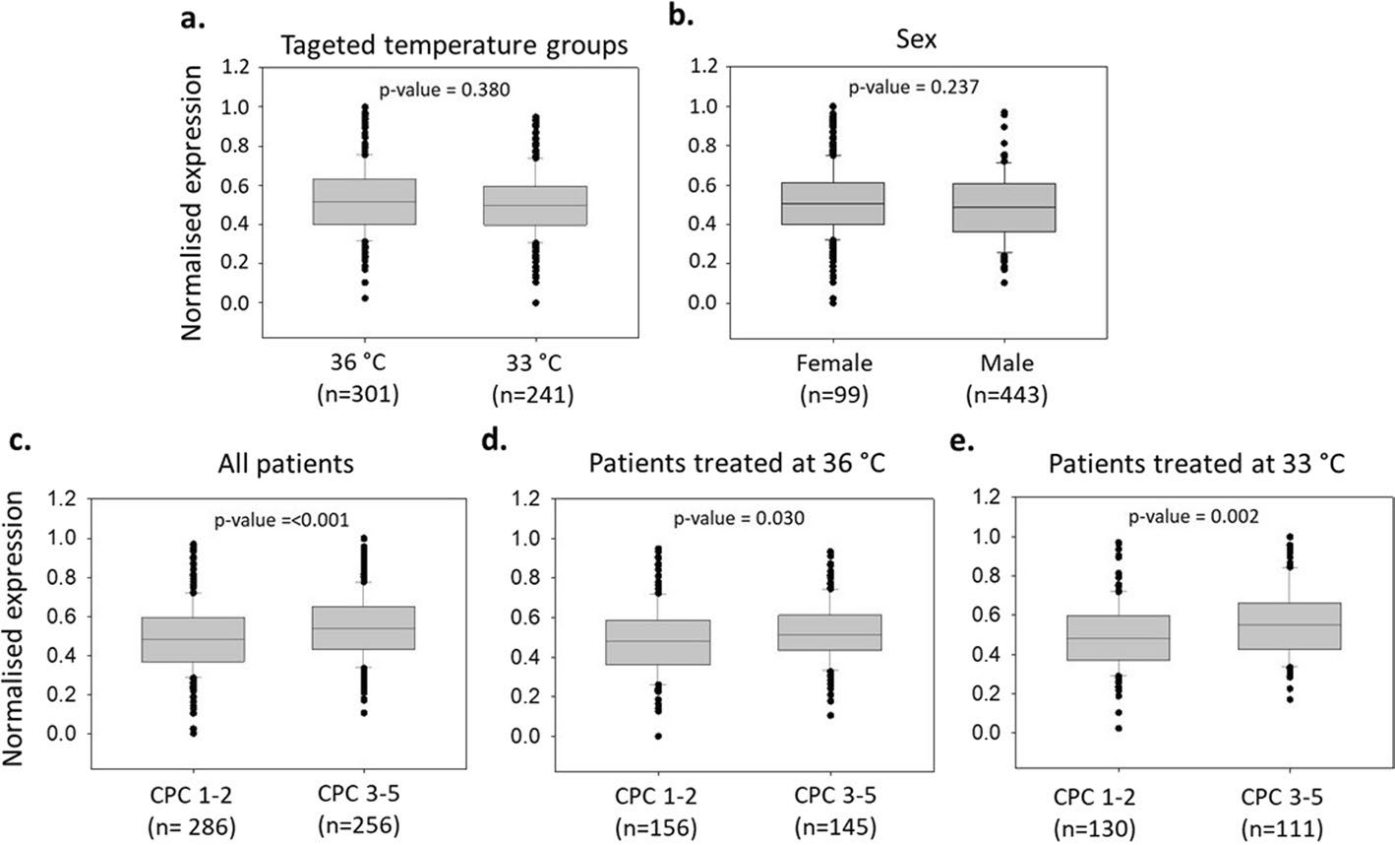
CircNFAT5 expression levels in 542 patients of the TTM-trial. CircNFAT5 levels were compared according to the temperature regimen and regardless of the neurological outcome **(a)**, between females and males **(b),** between good (CPC 1–2) and poor (CPC 3–5) neurological outcome regardless of the temperature regimen **(c),** and separately in patients treated at 36 °C or 33 °C **(d, e)**. The expression levels of circNFAT5 were normalized, log2 transformed and scaled. *p*-values are from Mann–Whitney *U* test

Higher levels of circNFAT5 in poor outcome patients (CPC 3–5) compared to good outcome patients (CPC 1–2) measured by qPCR (Fig. 2c) confirmed the results of the RNA-seq data (Table 1). This difference persisted when separating patients by temperature treatment at 33 °C or 36 °C (Fig. 2d, e). Targeted temperature regimen did not affect circNFAT5 levels, and males and females had comparable levels of circNFAT5 (Fig. 2a, b). These results were confirmed also when patients were dichotomized according to mRS score (Additional file 3: Fig. S5).

**Fig. 3:**
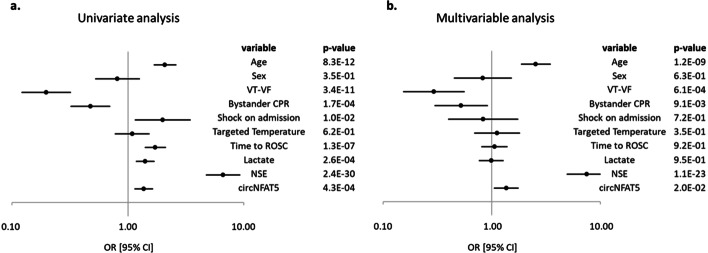
Logistic regression models to predict 6-month neurological outcome. Forest plots showing the odds ratio (OR) with ± 95% confidence interval [95% CI] for the prediction of 6-month neurological outcome in TTM-trial patients. **a** Univariate logistic regression analysis. **b** Multivariable logistic regression analysis

**Fig. 4:**
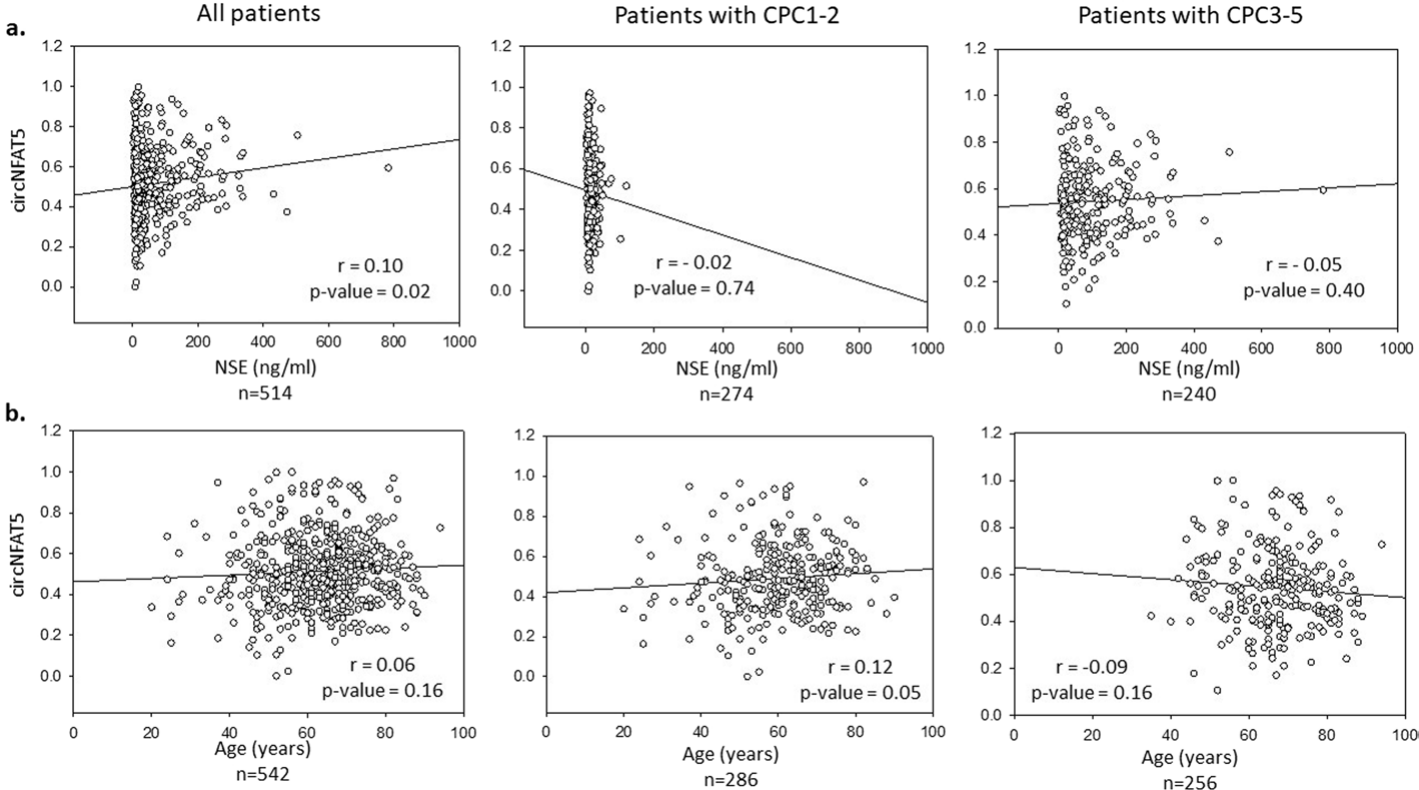
Correlation analyses of circNFAT5 with NSE and age. Scatter plots and linear regressions showing the correlations between circNFAT5, NSE (**a**) and age (**b**). Correlations were conducted considering all patients or patients dichotomized according to neurological outcome as CPC 1–2 and CPC 3–5. The expression levels of circNFAT5 used for these analyses were normalized, log2 transformed and scaled. circNFAT5 and NSE levels were measured 48 h after OHCA. Spearman correlation coefficients (r) and *p*-value are indicated in each plot

To assess the potential of circNFAT5 to predict the neurological outcome of patients in the TTM-trial 6 months after OHCA, we conducted univariate and multivariable logistic regression analyses using both CPC score (Fig. 3) and mRS score (Additional file 3: Fig. S6) to classify patients. Consistent with previous studies in this trial, the following parameters were included in the multivariable clinical model: age, sex, first monitored rhythm, bystander cardiopulmonary resuscitation (CPR), circulatory shock on admission, targeted temperature regimen, time from CA to ROSC, initial serum lactate levels and NSE levels at 48 h [33, 34]. As indicated in Fig. 3 and Additional file 3: Table S2, circNFAT5 was a univariate predictor of the neurological outcome and this prediction remained significant after adjustment with clinical parameters (univariate OR [95% CI]: 1.37 [1.15–1.63] and 1.39 [1.07–1.83] after adjustment). In the multivariable analysis, age, first monitored rhythm, bystander CPR and NSE levels were also independent predictors of neurological outcome (Fig. 3b and Additional file 3: Table S2). The results were also confirmed when using mRS instead of CPC as dichotomization score (Additional file 3: Fig. S6). Furthermore, correlation analyses showed no association between circNFAT5, NSE and age (Fig. 4) both considering all patients (*r* = 0.10 and *r* = 0.06, respectively) or only patients with good (*r* = 0.02 and *r* = 0.12, respectively) or poor (*r* = 0.05 and *r* = 0.09, respectively) neurological outcome.

**Fig. 5:**
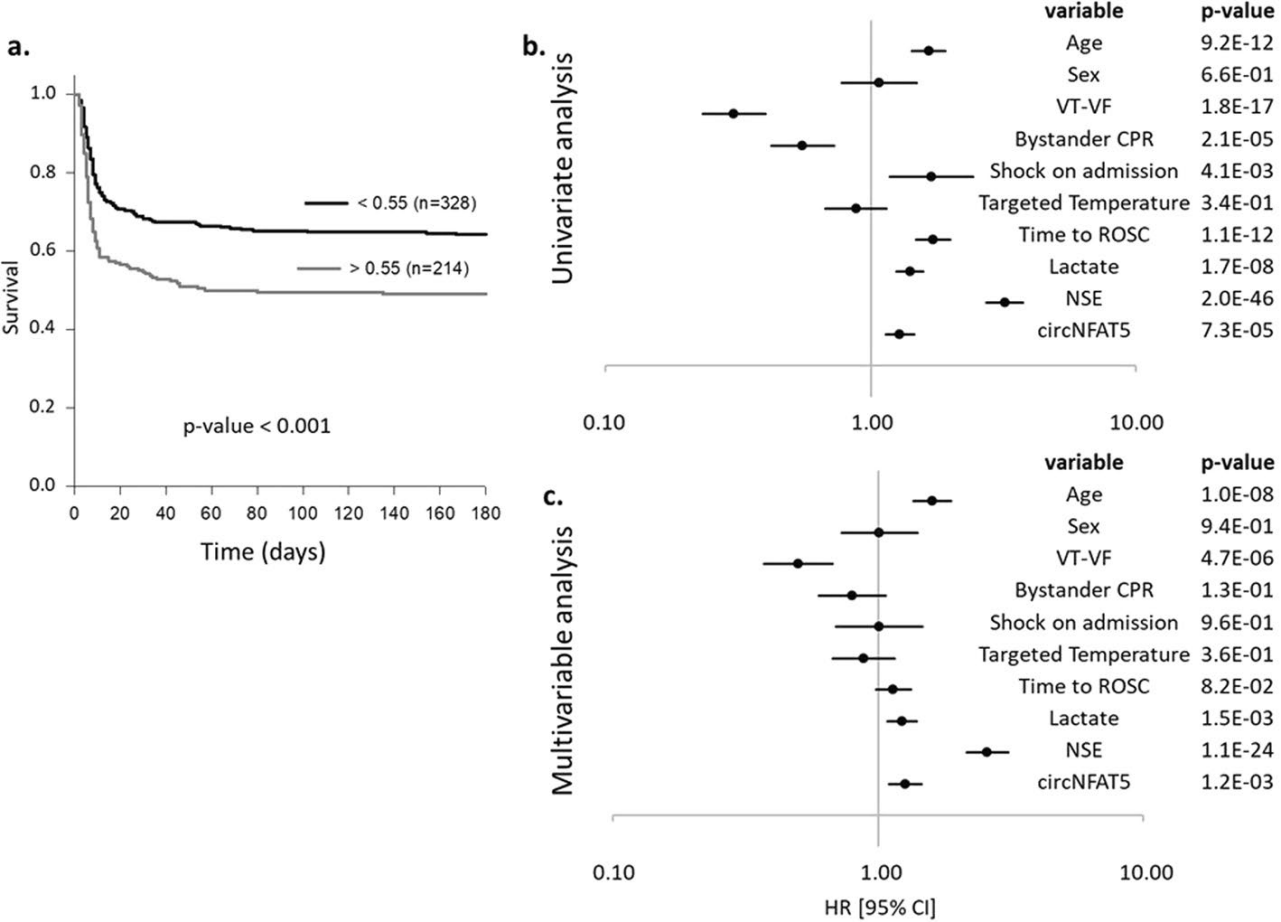
Survival analyses in TTM-trial patients at 6 months. 6-month survival of TTM-trial patients using Kaplan–Meier curves and Cox proportional hazards. **a** Kaplan–Meier curves using the Youden’s index as cut-off value. **b** Univariate Cox proportional hazards for 9 independent variables and circNFAT5. **c** Multivariable Cox proportional hazards model. Data are presented as hazards ratio (HR) with ± 95% confidence interval [95% CI] and *p*-values are indicated for each variable

Kaplan–Meier survival curves and Cox proportional hazards models were then used to estimate the ability of circNFAT5 to predict 6-month survival. Kaplan–Meier survival curves generated using the Youden’s index as cut-off value indicate a higher chance of survival (*p* < 0.001) in patients with expression levels of circNFAT5 below 0.55 (Fig. 5a). In Cox proportional hazards models, a HR [95% CI] of 1.29 [1.14–1.46] indicated that the increase of circNFAT5 was associated with a higher risk of death at 6 months (Fig. 5b and Additional file 3: Table S3) and this association remained significant after adjustment with demographic and clinical parameters (HR [95% CI]: 1.31 [1.13–1.52]; Fig. 5c and Additional file 3: Table S3). After adjustment, age, first monitored rhythm, lactate and NSE levels were also independent predictors of 6-month survival (Fig. 5c).

The incremental value of circNFAT5 to predict neurological outcome and survival was assessed using the Akaike Information Criterion (AIC). For neurological outcome prediction using CPC score, a decrease of AIC was observed when integrating circNFAT5 in the model (LRT *p*-value = 0.015; Hosmer–Lemeshow *p*-value < 2.22E-16). This was associated with an IDI of 0.005 (*p* = 0.15), a NRI of 0.27 (*p* = 0.001) and an AUC of 0.91 (Table 4). Similar results were obtained for neurological outcome prediction using mRS score (Additional file 3: Table S4).

**Table 4:** CircNFAT5 performance to predict patient outcome in TTM cohort

Neurological outcome	*AIC*	*HL_p*	*AUC*	*lr_p*	*NRI*	*NRI_p*	*IDI*	*IDI_p*
*Basal model*	433	< 2.22E-16	0.91	–	–	–	–	–
*Basal model* + *circNFAT5*	429	< 2.22E-16	0.91	1.49E-02	0.27	1.37E-03	5.34E-03	0.15

Survival	*AIC*	*GB_p*	*C_idx*	*lr_p*
Basal model	2373	1.57E-03	0.85	–
Basal model + circNFAT5	2362	1.71E-03	0.85	3.91E-04

Incremental value of circNFAT5 to predict neurological and survival in TTM-trial patients. The basal model included age, sex, first monitored rhythm, bystander cardiopulmonary resuscitation (CPR), circulatory shock on admission, targeted temperature regimen, time from CA to ROSC, initial serum lactate levels and NSE levels at 48 h. *AIC* Akaike Information Criterion, *HL* Hosmer–Lemeshow, *AUC* area under the curve, *GB* Gronnesby and Borgan, *C_idx* Harrell’s C-index, *IDI* integrated discrimination improvement, *IDI_p* integrated discrimination improvement *p*-value, *lr_p* likelihood ratio test *p*-value, *NRI* net reclassification improvement, *NRI_p* net reclassification improvement *p*-value

CircNFAT5 was able to improve the survival model as attested by a decrease of AIC (LRT *p*-value = 3.91E-04; Gronnesby and Borgan *p*-value = 8.62E-74), and this was associated with a C-index of 0.85 (Table 4).

## Discussion

The present study aimed to identify and validate the prognostic potential of circulating circRNAs after OHCA. In a discovery phase using whole transcriptome sequencing, one circRNA named circNFAT5 (hsa-circ-0006845) was identified and selected for validation of its prognostic ability in the TTM-trial. Our study showed that patients with elevated circulating levels of circNFAT5 48 h after OHCA were at higher risk of poor neurological outcome and death.

Although circRNAs are believed to have many regulatory functions within the cells [35–37], the mode of action of most of them including circNFAT5, remains to be determined. It is believed that different cell types can express clusters of genes in response to diverse insults [38, 39]. Therefore, the study of neighboring genes could be an indicator of the function of a specific circRNA. CircNFAT5 is located in the last coding exon of the transcription factor NFAT5 which is expressed in several tissues such as skeletal muscle, heart, brain and peripheral blood leukocytes [40–43]. Its activation after stress and its tissue distribution makes it appealing and provides a potential link between the heart, brain and leukocytes, with all three of them contributing to clinical outcome after cardiac arrest. Importantly, leukocytes are the cells where the measurements of circNFAT5 are taking place and we have observed that circNFAT5 was expressed in lymphocytes and monocytes but was not detectable in serum (Additional file 2). Additionally, several studies have previously reported that NFAT5 plays a key role in inflammatory processes of pathologies associated with cardiovascular diseases such as hypertension, atherosclerosis and diabetes mellitus [44–47]. Therefore, it would be interesting to investigate a hypothetical involvement of circNFAT5 in inflammatory pathways associated with CA and its clinical outcome and sequelae, in relationship with NFAT5. In addition to this, we observed a low correlation between circNFAT5 and NSE levels (Fig. 4) which suggests an involvement of circNFAT5 in processes occurring after OHCA other than brain injury. This supports the incremental prognostic potential of circNFAT5 to be used in combination with established markers of OHCA such as NSE. Although NSE or more recently discovered biomarkers such as Nfl, are good predictors of neurological outcome after CA, novel biomarkers reflecting other pathways (than neuronal death) involved in outcome after CA could provide some incremental predictive value to ensure a maximal prediction accuracy. Along with that, it would be interesting to conduct further post hoc analysis to define whether circNFAT5 can better predict the outcome of specific subpopulations of patients, such as patients with shock on admission. However, this requires larger population than the TTM cohort included in the present study. This study has the strength of being a predefined substudy of a large multicenter clinical trial on OHCA. Blood collection, processing and storage was performed homogenously in each center according to standard operating procedures implemented and validated by our central biobank. Furthermore, the measurements of circNFAT5 were conducted in a single laboratory according to pre-established protocols [25], limiting the inter-laboratory variability of sample processing. All together, these measures ensure sample quality and robustness of the results. The present study also has some limitations. Firstly, the predictive value of only one circRNA from our discovery study has been extensively tested and reported, which does not exclude the presence of other circRNAs that can aid in the prognostication of OHCA patients. Combination of several circRNAs in prediction models remains to be tested. Secondly, the cellular origin of circNFAT5 was not accurately determined and neither was the mechanism that links circNFAT5 with outcome after OHCA. Finally, circNFAT5 was measured at a single time point, 48 h after OHCA, and it is unknown whether it can be detected at an earlier stage. Despite these limitations, our study is the first to highlight the potential and unexplored biomarker ability of circRNAs for outcome prediction after OHCA and therefore represents the starting point for future biomarker and functional studies focusing on the role played by circRNAs in CA pathophysiology.

## Conclusions

In the present study, elevated circulating levels of the circular RNA, circNFAT5, measured 48 h after ROSC were associated with a higher risk of poor neurological outcome and death after OHCA. The incremental predictive value of circNFAT5 may emanate from its association with other post-cardiac arrest mechanisms than neurological damage, such as inflammation. However, the exact functional association between cNFAT5 and outcome after OHCA remains to be determined.

## Supplementary Information

**Additional file 1.** Sequencing results of the five candidate circRNAs.

**Additional file 2.** Expression profiles of circNFAT5 in different blood compartments from samples of 3 volunteers.

**Additional file 3.** Supplemental methods and results.

## Data Availability

The circRNAs metadata in TTM can be accessed using the GEO code GSE197764.

## Abbreviations

AIC: Akaike Information Criterion
CA: Cardiac arrest
cDNA: Complementary DNA
CI: Confidence interval
circNFAT5: Circular NFAT5
circRNA: Circular RNA
COPD: Chronic obstructive pulmonary disease
CPC: Cerebral performance category
CPR: Cardiopulmonary resuscitation
HR: Hazard ratio
IBBL: Integrated Biobank of Luxembourg
IDI: Integrated discrimination improvement index
LRT: Likelihood-ratio test
mRS: Modified Rankin Score
ncRNA: Non-coding RNA
NFL: Neurofilament light chain
NRI: Net reclassification improvement
NSE: Neuron-specific enolase
OHCA: Out-of-hospital cardiac arrest
OR: Odds ratio
PEA: Pulseless electrical activity
qPCR: Quantitative PCR
RNA-seq: RNA sequencing
ROSC: Return of spontaneous circulation
RT: Reverse transcription
TTM: Target temperature management trial
VF: Ventricular fibrillation
VT: Ventricular tachycardia
