# Correction: Circular RNAs to predict clinical outcome after cardiac arrest

**DOI:** 10.1186/s40635-022-00477-0

**Published:** 2022-11-09

**Authors:** Francesca M. Stefanizzi, Lu Zhang, Antonio Salgado-Somoza, Josef Dankiewicz, Pascal Stammet, Christian Hassager, Matthew P. Wise, Hans Friberg, Tobias Cronberg, Alexander Hundt, Jesper Kjaergaard, Niklas Nielsen, Yvan Devaux

**Affiliations:** 1 Cardiovascular Research Unit, Department of Population Health, Luxembourg Institute of Health, 1A-B Rue Edison, 1445 Strassen, Luxembourg; 2 Department of Cardiology, Clinical Sciences, Lund University and Skane University Hospital, 221 85 Lund, Sweden; 3 Department of Intensive Care Medicine, Centre Hospitalier de Luxembourg, 1210 Luxembourg, Luxembourg; 4 Department of Cardiology B, The Heart Centre, Rigshospitalet University Hospital, 2100 Copenhagen, Denmark; 5 Department of Intensive Care, University Hospital of Wales, Cardiff, CF14 4XW UK; 6 Department of Anesthesia and Intensive Care, Clinical Sciences, Lund University and Skane University Hospital, 221 85 Malmö, Sweden; 7 Department of Neurology and Rehabilitation Medicine, Clinical Sciences, Lund University and Skane University Hospital, 221 85 Lund, Sweden; 8 Integrated BioBank of Luxembourg, Luxembourg Institute of Health, Dudelange, Luxembourg; 9 Department of Anesthesia and Intensive Care, Clinical Sciences, Lund University and Helsingborg Hospital, 25187 Lund, Sweden; 10 Department of Life Sciences and Medicine, Faculty of Science, Technology and Medicine, University of Luxembourg, 4365 Esch-Sur-Alzette, Luxembourg

## Correction: Intensive Care Medicine Experimental (2022) 10:41 https://doi.org/10.1186/s40635-022-00470-7

Following publication of the original article [1], the authors have updated the funding information of their article as requested by their funding institution.

Namely, the funding declaration has been corrected to the following:

"This work is supported by the National Research Fund of Luxembourg (Grants # C14/BM/8225223 and C17/BM/11613033 to YD), the Ministry of Higher Education and Research of Luxembourg, and the Heart Foundation - Daniel Wagner. FMS received a fellowship from the National Research Fund of Luxembourg for this work (Grant # C17/BM/11613033). This work is also supported by independent research grants from nonprofit or governmental agencies (the Swedish Research Council [Vetenskapsrådet], Swedish Heart–Lung Foundation, Stig and Ragna Gorthon Foundation, Knutsson Foundation, Laerdal Foundation, Hans-Gabriel and Alice Trolle-Wachtmeister Foundation for Medical Research, and Regional Research Support in Region Skåne) and by governmental funding of clinical research within the Swedish National Health Service."

The authors thank you for reading and apologize for any inconvenience caused.
