# Supplementary material for: Circular RNAs to predict clinical outcome after cardiac arrest
Source: Intensive Care Med Exp. 2022 Oct 28;10:41. doi: 10.1186/s40635-022-00470-7 (PMC9613847; doi:10.1186/s40635-022-00470-7)

# Results sequencing 5 circRNAs

## circNFAT5

### Primers

>circNFAT5-H-S1

AGATTGATTTGCTTGTTTCa

>circNFAT5-H-AS1

TGAGAAAGAAGTGTTGTC

### Full sequence circNFAT5

>hg19_hub_77_jeck_circRNAs range=chr16:69729039-69729282 5'pad=0 3'pad=0 strand=+ repeatMasking=none

ATGCCAGAGAATTCTCCACTGGCATCCTCTATAAACACCAACCAGAACATCGAAAAGATTGATTTGCTTGTTTCATTGCAAAACCAAGGGAACAACTTGACTGGCTCCTTTTAACTGGATATACTGTAGTCAGCTTTTAACCTCTGGACCAGCTACATTGCCTGATCAGTTGATGGCCATAAGTCAGCCAGGCCAACCACAAAACGAGGGCCAGCCACCTGTGACAACACTTCTTTCTCAGCAA

### Sequence amplified

>circNFAT5_S1

CGGACACTTGMTGGCTCCTTTTAACTGGATATACTGTAGTMGCTTTTAACCTCTGGACCAGCTACATTGCCTGATCAGTTGATGGCCATAAGTCAGCCAGGCCAACCACAAAACGAGGGCCAGCCACCTGTGACAACACTTCTTTCTCAA


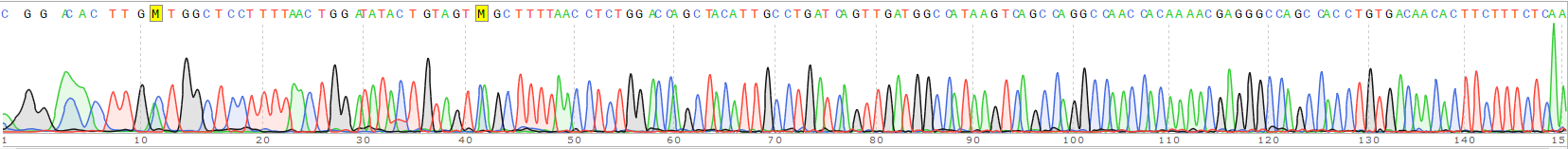


>circNFAT5_AS1

TAGATTGATTTGCTTGTTTCATTGCAAAACCAAGGGAACAACTTGACTGGCTCCTTTTAACTGGATATACTGTAGTCAGCTTTTAACCTCTGGWCCAGCTACATTGCCKATCAGTTGAKGCCATAAGTCAGCCAGGCCAACCACAAAACGAGGCAGCCACT


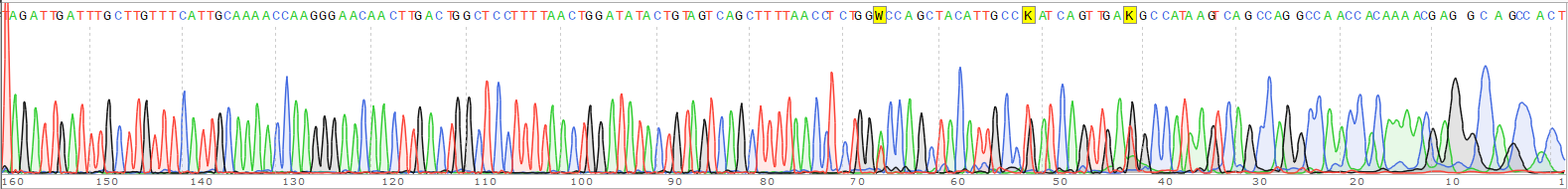


## CircDNM2

### Primers

>circDNM2-H-S1

AGAGGAGACAGAGCGAAT

>circDNM2-H-AS1

CGTAAGTCCTTCTCGTCAA

### Full sequence circDNM2

>hsa_circ_0049335|chr19:10906047-10909248+|NM_001005360|DNM2

AGAGGAGACAGAGCGAATCGTCACCACTTACATCCGGGAACGGGAGGGGAGAACGAAGGACCAGATGGAGTTTGACGAGAAGGACTTACGACGGGAGATCAGCTATGCCATTAAGAACATCCATGGAGTCAGGACGGGGCTCTTCACCCCCGACATGGCCTTTGAAGCCATTGTGAAAAAACAGATTGTAAAACTCAAAGAGCCGAGTTTGAAGTGTGTTGATCTCGTGGTCTCAGAGCTGGCCACGGTCATAAAAAAGTGTGCCGAGAAGCTCAGTTCCTACCCCCGGTTGCG

### Sequence amplified

>PCRcic_DNM2_H_S1Mix2_AS_100717_E02

TGCTTCTCCGACGGGAGGGGAGACGAAGGACCAGATGGAGTTTGACGAGAAGGACTTACGA


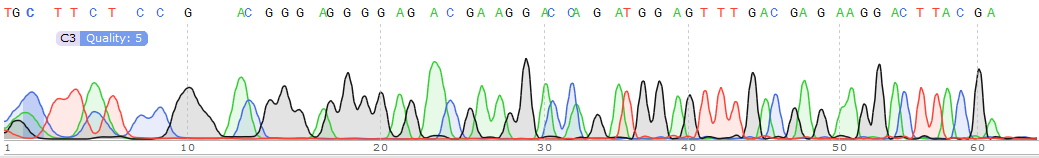


>PCRcic_DNM2_H_AS1Mix2_AS_100717_F02

TAGAGGAGACAGAGCGAATCGTCACCACTTACTCCGGGAACGGGAGGGAGAACGAAGACA


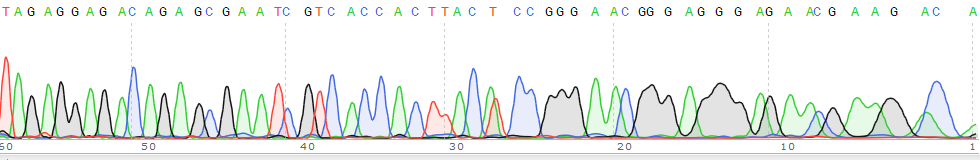


## CircFAM13b

### Primers

>hsa_circ_0001535_FAM13B-S1

ATTGTGATGGGGAAGGAT

>hsa_circ_0001535_FAM13B-AS1

GATATATGGGTGCTGGGT

### Full sequence circFAM13b

>hsa_circ_0001535_FAM13B:331-165

ATTCATTGTGATGGGGAAGGATCTAATAACCAGATTGATATTGCTGATGATATTATTAATGCCAGTGAAAGTAACAGAGACTGTTCAAAACCTGTGGCTAGCACTAATTTAGACAATGAAGCTATGCAGCAAGATTGTGTATTTGAGAATGAAGAAAATACCCAGCACCCATATATCTCCCATCAGCATCCTACCAGCCTCTACAGATATTTTAGAAAGAACAATTAGAGCAGCTGTGGAACAGCACCTTTTTGATCTTCAGAGCAGCATAGATCATGATCTTAAGAATTTACAACAGCAAAGTGTGGTGTGTAATAATGAAGCAGAAAGT

### Sequence amplified

>circFAM13B-f_G02

ACGTATGWATTGCTGATGATATTATTAATGCMGTGAAAGTAACAGAGACTGTTCAAAACCTGTGGCTAGCACTAATTTAGACAATGAAGCTATGCAGCAAGATTGTGTATTTGAGAATGAAGAAAATACCCAGCACCCATATATCA


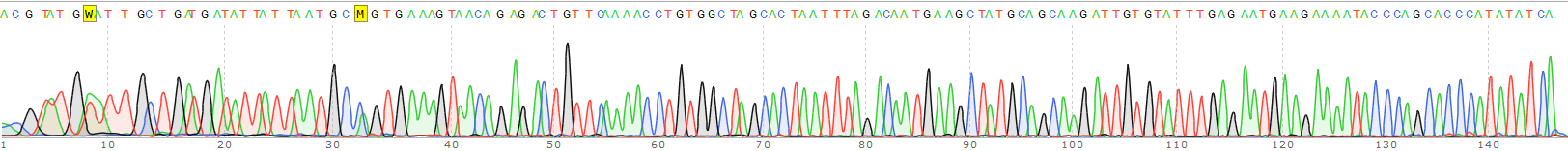


>circFAM13B-r_H02

TTATAGTGTTAGCCACTGGAGTGATCGGTCTCAGTACTTTCACTTGCACCTATTTTTCATCAGCAATTGTWMCTATATTGTGATGGGGAAGGATCTAATAACCAGATTGATATTGCTGATGATATTATTAATGCCAGTGAAAGTAACAGAGACTGTTCAAAACCTGTGGCTAGCACTAATTTAGACAATGAAGCTATGCAGCAAGATTGTGTATTKRAT


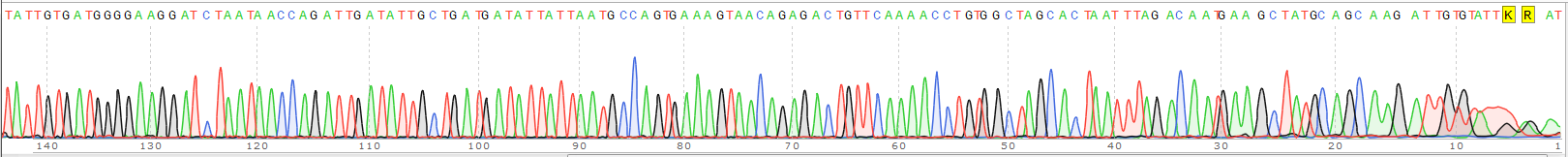


## CircAGO2

### Primers

>hsa_circ_0002965_AGO2-S1

TTAACAGGGAAATCGTGGAA

>hsa_circ_0002965_AGO2-AS1

AGGTGCAAGTGCTTGTC

### Full sequence circAGO2

>hsa_circ_0002965_AGO2:314-157

TATCAAGCCAGAGAAGTGCCCGAGGAGAGTTAACAGGGAAATCGTGGAACACATGGTCCAGCACTTTAAAACACAGATCTTTGGGGATCGGAAGCCCGTGTTTGACGGCAGGAAGAATCTATACACAGCCATGCCCCTTCCGATTGGGAGGGACAAGCACTTGCACCTCCTGCGCCGCCGCCCCCCATCCAAGGATATGCCTTCAAGCCTCCACCTAGACCCGACTTTGGGACCTCCGGGAGAACAATCAAATTACAGGCCAATTTCTTCGAAATGGACATCCCCAAAATTGACATCTATCATTATGAATTGGA

### Sequence amplified

>circAgo2-f_G03

TCGGTCMGCMTTTAACACAGATCTTTGGGGATCGGAAGCCCGTGTTTGACGGCAGGAAGAATCTATACACAGCCATGCCCCTTCCGATTGGGAGGGACAAGCACTTGCACCTA


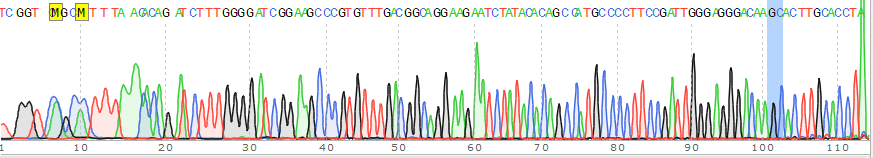


>circAgo2-r_G03

TTTAACAGGGAAATCGTGGAACACATGGTCCAGCACTTTAAAACACAGATCTTTGGGGATCGGAAGCCCGTGTTTGACGGCAGGAAGAATCTATACACAGCCATGCCCTCG


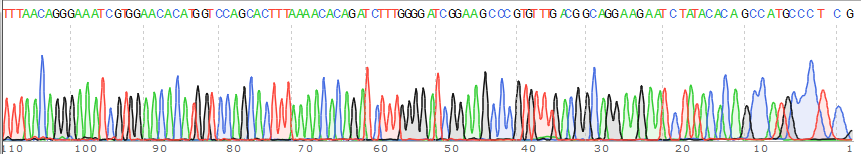


## CircDLG1

### Primers

>hsa_circ_0068702_DLG1-S1

AAACACCAACTTACCCAAC

>hsa_circ_0068702_DLG1-AS1

GTATTCTCAGCAGGGACT

### Full sequence circDLG1

>hsa_circ_0068702_DLG1:153-76

CATCCTACCCACCATACCACAGGCAAATCCTCCCCCAGTACTGGTCAACACAGATAGCTTGGAAACACCAACTTACCCAACAGAAGCTGTTCTTCCCTCTCCTCCCACTGTCCCTGTGATCCCTGTCCTGCCAGTCCCTGCTGAGAATACTGT

### Sequence amplified

>circDLG1_s1

TYATCTCCACTGTCCTGTGATCCCTGTCCTGCCAGTCCCTGCTGAGAATACA


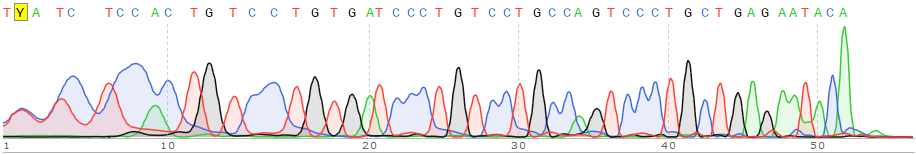


>circDLG1_as1

TAAACACCAACTTACCCAACRGARGSTGKCTSCCTSTCCTCCCRCTSCTACGATGCTGTTACGT


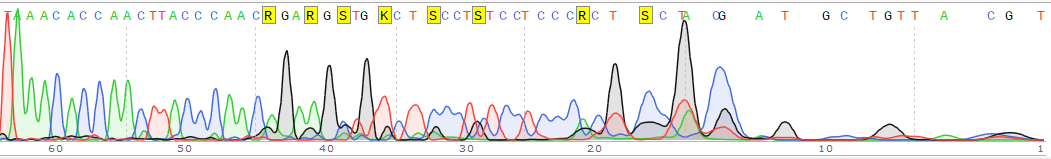

Supplement: Supplementary file 1 — Additional file 1. Sequencing results of the five candidate circRNAs. [file 40635_2022_470_MOESM1_ESM.docx]
