# Supplementary material for: Circular RNAs to predict clinical outcome after cardiac arrest
Source: Intensive Care Med Exp. 2022 Oct 28;10:41. doi: 10.1186/s40635-022-00470-7 (PMC9613847; doi:10.1186/s40635-022-00470-7)
Supplement: Supplementary file 2 — Additional file 2. Expression profiles of circNFAT5 in different blood compartments from samples of 3 volunteers. [file 40635_2022_470_MOESM2_ESM.pptx]

## Slide 1
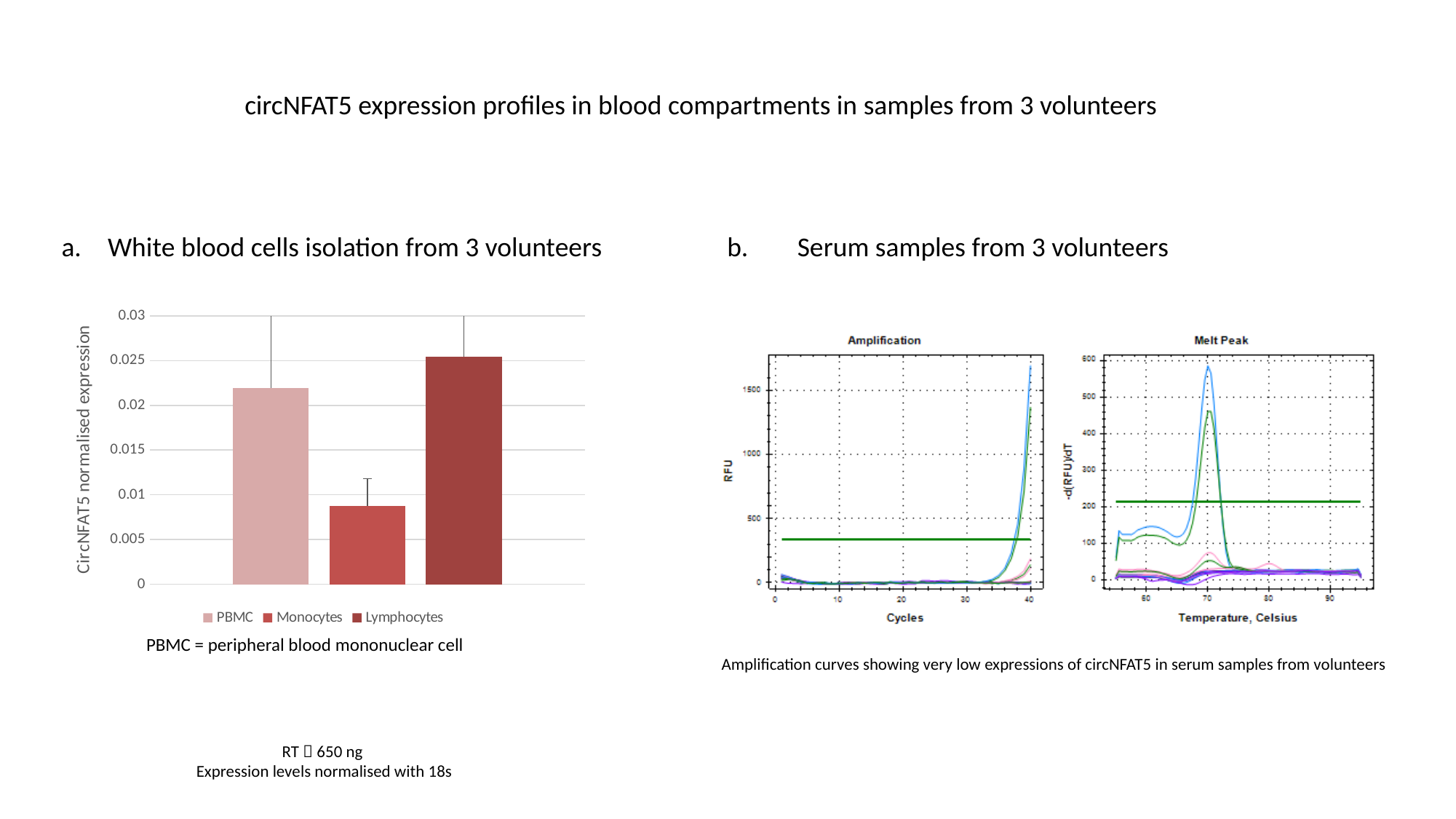

circNFAT5 expression profiles in blood compartments in samples from 3 volunteers
a.
White blood cells isolation from 3 volunteers
b.
Serum samples from 3 volunteers
### Chart
| Category | PBMC | Monocytes | Lymphocytes |
|---|---|---|---|
PBMC = peripheral blood mononuclear cell
Amplification curves showing very low expressions of circNFAT5 in serum samples from volunteers
RT  650 ng
Expression levels normalised with 18s
