# Supplementary material for: Circular RNAs to predict clinical outcome after cardiac arrest
Source: Intensive Care Med Exp. 2022 Oct 28;10:41. doi: 10.1186/s40635-022-00470-7 (PMC9613847; doi:10.1186/s40635-022-00470-7)
Supplement: Supplementary file 3 — Additional file 3. Supplemental methods and results. [file 40635_2022_470_MOESM3_ESM.docx]

Supplemental methods

RNA extraction

Total RNA was extracted from PAXgene^TM^ Blood RNA tubes using the PAXgene^TM^ Blood miRNA Kit (Qiagen), according to the manufacturers’ protocol, followed by spectrophotometric quantification.

RNA sequencing (RNA-seq)

A strand-specific RNA-Seq library was prepared using the Ovation Human Blood RNA-Seq Library Systems (NuGEN Technologies, San Carlos, USA) from 46 human whole blood samples from TTM-trial. The sequencing was performed using the HiSeq platform (Illumina, San Diego, CA) with 2 x 50 bp. After trimming the adapters, quality control of the RNA-seq data was performed using the FastQC tool (https://www.bioinformatics.babraham.ac.uk/projects/fastqc/). The reads were aligned to the human reference genome (GRCh38) using STAR with default set-up (34).DCC was used with default set-up for circRNA identification (35) and DESeq2 (36) to identify differentially expressed circRNAs. The circRNAs detected with at least 2 reads in at least half samples of CPC1 or CPC5 group were kept in differential expression analysis. The circRNAs with p-value < 0.05 and log2 fold change > 0.5 or < -0.5 were kept for candidate selection and validation by quantitative PCR (qPCR).

A volcano plot was generated using ggplot (37) to show the –log 10 p-values versus log2 fold change of detected circRNAs. A UMAP (Uniform Manifold Approximation and Projection) plot (38) was used to show the separation of CPC1 and CPC5 groups with the normalized counts of differentially expressed circRNAs. The heatmap of differentially expressed circRNAs was generated using pheatmap R package ([https://CRAN.R-project.org/package=pheatmap](about:blank)) with Euclidean distance.

Reverse transcription

Reverse transcription (RT) was performed on 300 ng of RNA per sample with the SuperScript™ II Reverse Transcriptase kit following the manufacturer’s protocol (ThermoFisher scientific cat n. 18064071) with the addition of random primers (Invitrogen, P/N 58875) and dNTPs (Invitrogen, cat n.18427088). Controls without reverse transcriptase allowed verifying the absence of genomic DNA amplification in each RT series. The resulting cDNA was diluted 10-fold before measuring the expression levels of circNFAT5 by qPCR.

Quantitative PCR

Pairs of divergent PCR primers were designed using Beacon Designer software version 8.0 (Premier Biosoft) for circRNAs engaged in qPCR experiments. Divergent primers allow a specific amplification of circular and not linear RNAs. Primers for circNFAT5 were as follows: circNFAT5-H-S1: AGATTGATTTGCTTGTTTC and circNFAT5-H-AS1: TGAGAAAGAAGTGTTGTC. The optimal annealing temperature selected for circNFAT5 was 58 °C with a PCR efficiency of 93.9%. PCR product sequencing and melt curve analysis confirmed the specificity of the amplification. Each PCR plate contained an internal standard calibrator and a cDNA pool originated from five PAXgene^TM^ Blood RNA tubes of CA patients. The internal standard calibrator allowed interplate variability to be corrected. Furthermore, each plate contained appropriate negative controls in order to check for potential mastermix or sample contamination. Quantitative PCRs were performed using CFX96 thermocycler (Bio-Rad). SF3a1 was used as housekeeping gene for normalisation. The expression values were determined using the relative quantification method (ΔΔCt) with CFX Maestro 1.1 software (Bio-Rad), followed by log2 transformation and scaling.

RNaseR treatment

The circularity of 5 circRNAs was confirmed in PAXgene blood samples of CA patients. Eight samples were selected for the RNase R treatment: four samples from patients with a CPC1 and four from patients with a CPC5. 500 ng of total RNA were used for the treatment with RNaseR enzyme (Epicentre; cat n. RNR07250) and another 500 ng was used for the mock. The RNaseR treatment was performed following the manufacturer’s protocol. Thus, RT and qPCRs were performed following the same previously described protocol. SF3a1 is a linear transcript and therefore considered as a negative control of the experiment while, MICRA, a known circular RNA, was used as a positive control to confirm circRNAs circularity. RNase R resistance was quantified as the average of the 8 samples reported as a percentage of (2^-Cq _RNaseR)/(2^-Cq_mock) for each candidate circRNA.

Supplemental results

Supplemental tables

### Supplemental Table 1. Demographic and clinical characteristics of TTM cohort using mRS score.

| **Characteristics** | **Outcome patients** | | **p-value** |
| --- | --- | --- | --- |
|  | **mRS 0-3** | **mRS 4-6** |  |
|  | **n=289** | **n=253** |  |
| **Age, years** | 60 (20-90) | **68 (35-94)** | **<0.001** |
| **Sex** |  |  | 0.464 |
| Male | 240 (83%) | 203 (80.2%) |  |
| Female | 49 (17%) | 50 (19.8%) |  |
| **Co-morbidities** |  |  |  |
| Hypertension | 105 (36.3%) | 120 (47.4%) | **0.032** |
| Diabetes mellitus | 30 (10.4%) | 45 (17.8%) | **0.044** |
| Heart failure | 7 (2.4%) | 20 (7.9%) | **0.008** |
| COPD | 19 (6.6%) | 30 (11.9%) | **0.047** |
| **First monitored rhythm** |  |  | **<0.001** |
| VF or non-perfusing VT | 262 (90.7%) | 168 (66.4%) |  |
| Asystole or PEA | 17 (5.9%) | 77 (30.4%) |  |
| ROSC after bystander defibrillation | 7 (2.4%) | 1 (0.4%) |  |
| unknown | 3 (1%) | 7 (2.8%) |  |
| **Witnessed arrest** | 265 (91.7%) | 220 (87%) | 0.1 |
| **Bystander CPR** | 234 (81%) | 163 (64.4%) | **<0.001** |
| **Time from CA to ROSC, min** | 20 (0 - 160) | 30 (0 - 170) | **<0.001** |
| **Initial serum lactate (mmol/l)** | 5 (1 - 20) | 7 (1 - 21) | **<0.001** |
| **NSE 48h after ROSC (ng/ml)** | 15 (2.5 - 119.1) | 64.8 (3.1 - 782) | **<0.001** |
| **Shock on admission** | 25 (8.7%) | 36 (14.2%) | 0.056 |

Demographic and clinical characteristics of 542 patients of the TTM-trial according to neurological outcome established with mRS score. Same analyses as described in Table 3.

### Supplemental Table 2. Univariate and multivariable logistic regression analyses to predict 6-month neurological outcome

|  | **Univariate logistic regression** | | | |
| --- | --- | --- | --- | --- |
|  | *OR* | *Lower 95% CI* | *Upper 95% CI* | *p-value* |
| *Age* | 2.00 | 1.65 | 2.46 | 8.3E-12 |
| *Sex* | 0.81 | 0.52 | 1.25 | 3.5E-01 |
| *VT-VF* | 0.20 | 0.12 | 0.32 | 3.4E-11 |
| *Bystander CPR* | 0.48 | 0.32 | 0.70 | 1.7E-04 |
| *Shock on admission* | 1.99 | 1.16 | 3.49 | 1.0E-02 |
| *Targeted Temperature* | 0.92 | 0.65 | 1.29 | 6.2E-01 |
| *Time to ROSC* | 1.72 | 1.41 | 2.12 | 1.3E-07 |
| *Lactate* | 1.39 | 1.17 | 1.66 | 2.6E-04 |
| *NSE* | 7.50 | 5.40 | 10.78 | 2.4E-30 |
| *circNFAT5* | 1.37 | 1.15 | 1.63 | 4.3E-04 |
|  |  |  |  |  |
|  | **Multivariable logistic regression** | | | |
|  | *OR* | *Lower 95% CI* | *Upper 95% CI* | *p-value* |
| *Age* | 2.42 | 1.84 | 3.25 | 1.2E-09 |
| *Sex* | 0.86 | 0.46 | 1.60 | 6.3E-01 |
| *VT-VF* | 0.31 | 0.15 | 0.60 | 6.1E-04 |
| *Bystander CPR* | 0.46 | 0.26 | 0.82 | 9.1E-03 |
| *Shock on admission* | 0.87 | 0.41 | 1.84 | 7.2E-01 |
| *Targeted Temperature* | 0.79 | 0.48 | 1.30 | 3.5E-01 |
| *Time to ROSC* | 0.99 | 0.74 | 1.31 | 9.2E-01 |
| *Lactate* | 1.01 | 0.77 | 1.31 | 9.5E-01 |
| *NSE* | 9.17 | 6.08 | 14.48 | 1.1E-23 |
| *circNFAT5* | 1.39 | 1.07 | 1.83 | 2.0E-02 |

Univariate and multivariable logistic regression analyses reporting the odd ratio (OR) coefficients, 95% confidence intervals (Lower 95% CI and Upper 95% CI) and p-values for the different parameters tested.

### Supplemental Table 3. Univariate and multivariable Cox proportional hazards models to predict 6-month survival.

|  | **Univariate Cox proportional hazards** | | | |
| --- | --- | --- | --- | --- |
|  | *HR* | *Lower 95% CI* | *Upper 95% CI* | *p-value* |
| *Age* | 1.65 | 1.43 | 1.91 | 9.2E-12 |
| *Sex* | 0.93 | 0.67 | 1.29 | 6.6E-01 |
| *VT-VF* | 0.31 | 0.23 | 0.40 | 1.8E-17 |
| *Bystander CPR* | 0.55 | 0.42 | 0.73 | 2.1E-05 |
| *Shock on admission* | 1.70 | 1.18 | 2.43 | 4.1E-03 |
| *Targeted Temperature* | 0.88 | 0.68 | 1.15 | 3.4E-01 |
| *Time to ROSC* | 1.72 | 1.48 | 2.00 | 1.1E-12 |
| *Lactate* | 1.43 | 1.24 | 1.65 | 1.7E-08 |
| *NSE* | 3.54 | 3.09 | 4.07 | 2.0E-46 |
| *circNFAT5* | 1.29 | 1.14 | 1.46 | 7.3E-05 |
|  |  |  |  |  |
|  | **Multivariable Cox proportional hazards** | | | |
|  | *HR* | *Lower 95% CI* | *Upper 95% CI* | *p-value* |
| *Age* | 1.60 | 1.36 | 1.89 | 1.0E-08 |
| *Sex* | 1.01 | 0.73 | 1.41 | 9.4E-01 |
| *VT-VF* | 0.50 | 0.37 | 0.67 | 4.7E-06 |
| *Bystander CPR* | 0.80 | 0.60 | 1.06 | 1.3E-01 |
| *Shock on admission* | 1.01 | 0.70 | 1.47 | 9.6E-01 |
| *Targeted Temperature* | 0.88 | 0.68 | 1.15 | 3.6E-01 |
| *Time to ROSC* | 1.14 | 0.98 | 1.33 | 8.2E-02 |
| *Lactate* | 1.23 | 1.08 | 1.40 | 1.5E-03 |
| *NSE* | 2.58 | 2.15 | 3.09 | 1.1E-24 |
| *circNFAT5* | 1.27 | 1.10 | 1.46 | 1.2E-03 |

Univariate and multivariable Cox proportional hazards models reporting the hazard ratio (OR) coefficients, 95% confidence intervals (Lower 95% CI and Upper 95% CI) and p-values for the different parameters tested.

### Supplemental Table 4. CircNFAT5 performance to predict patient outcome in TTM cohort using mRS score.

| **Neurological outcome** | *AIC* | *HL_p* | *AUC* | *lr_p* | *NRI* | *NRI_p* | *IDI* | *IDI_p* |
| --- | --- | --- | --- | --- | --- | --- | --- | --- |
| *basal model* | 424 | < 2.22E-16 | 0.91 | - | - | - | - | - |
| *basal model + circNFAT5* | 420 | < 2.22E-16 | 0.91 | 0.015 | 0.285 | 7.97E-04 | 0.005 | 0.163 |

Incremental value of circNFAT5 to predict neurological outcome in TTM-trial patients using mRS score. Clinical model included in the analyses, analyses performed and abbreviations as in table 4.

Supplemental figures

Supplemental Figure 1. Study flow-chart of TTM-trial.

**
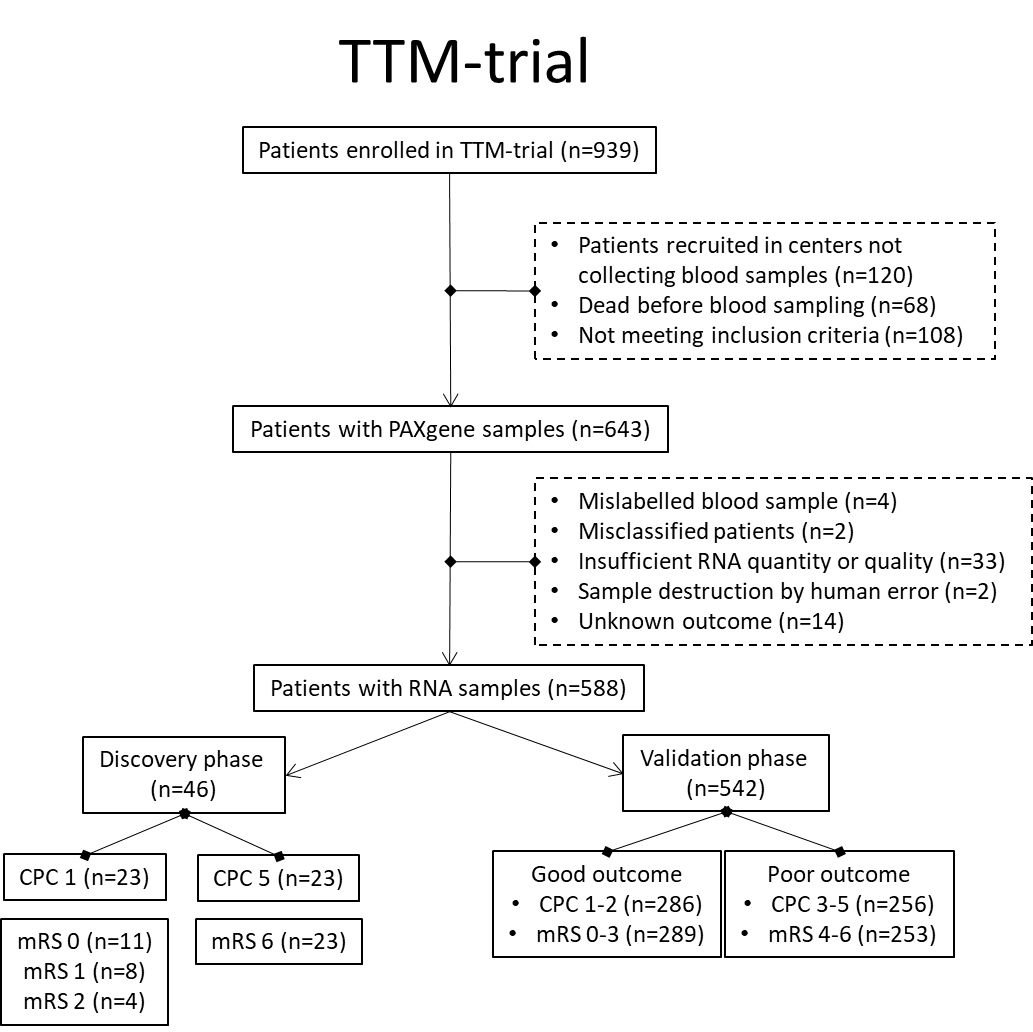
**

Abbreviations: TTM (Targeted Temperature Management); IBBL (International Biobank of Luxembourg); CPC (Cerebral Performance Category) mRS (modified Rankin Scale).

Supplemental Figure 2. PCR products of 5 circRNAs used for sequencing


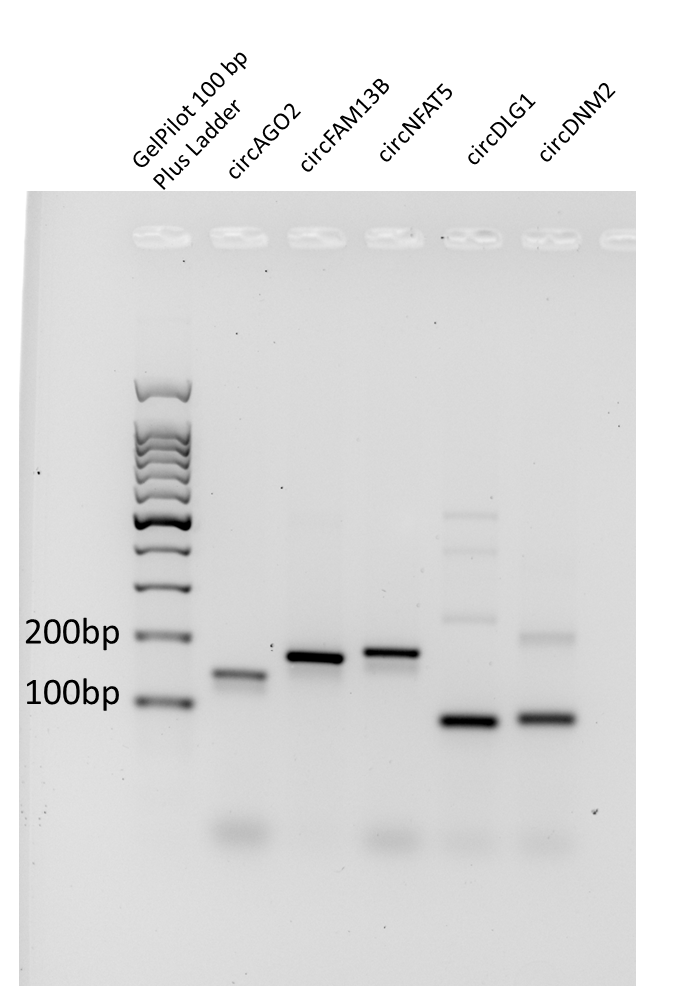


Electrophoretic gel showing the PCR products of the 5 circRNAs used for sequencing. The product size of each circRNA, circFAM13b (173 bp), circNFAT5 (185bp), circDLG1 (88 bp), circDNM2 (90 bp) and circAGO2 (139 bp) was confirmed. The electrophoretic run was performed on a 2% agarose gel. The ladder used was the GelPilot 100 bp Plus (cat. no. 239045).

Supplemental Figure 3. Confirmed circularity of the 5 circRNAs.


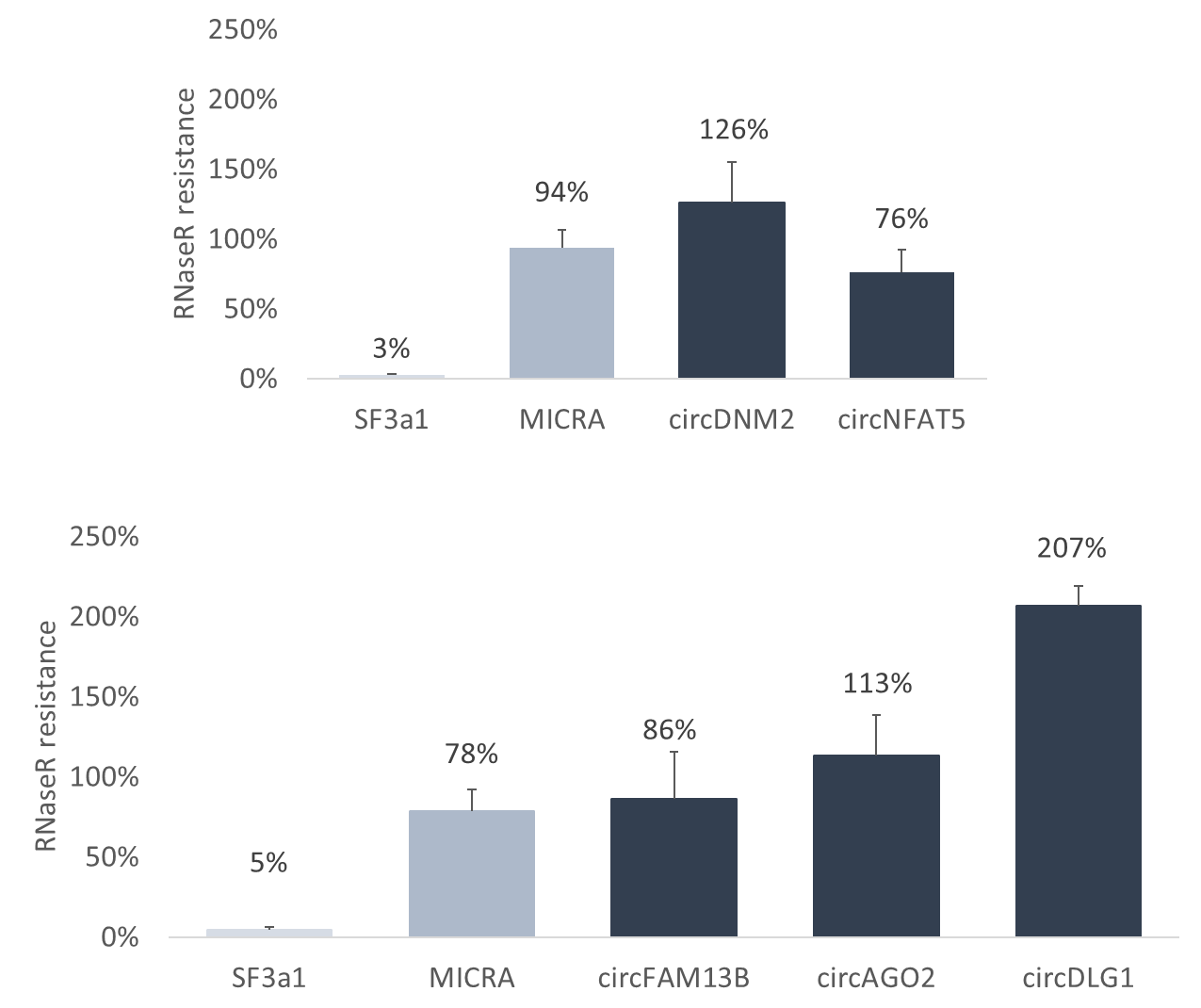


Bar chart showing resistance to RNAse R treatment of 5 circRNAs. The resistance to RNase R of circDNM2 and circNFAT5 was measured in a first set of 8 patients, whereas the resistance of circFAM13b, circAGO2 and circDLG1 was measured in a different set of patients and is therefore represented in a separate chart. SF3a1 linear RNA was used as a negative control of the experiment showing a 3-5% resistance to the treatment, while the circRNA MICRA (cZNF609) was used as positive control (78-94% resistance to the enzyme). Details on the protocol can be found in supplementary materials and methods section.

Supplemental Figure 4. Location and amplification circNFAT5.


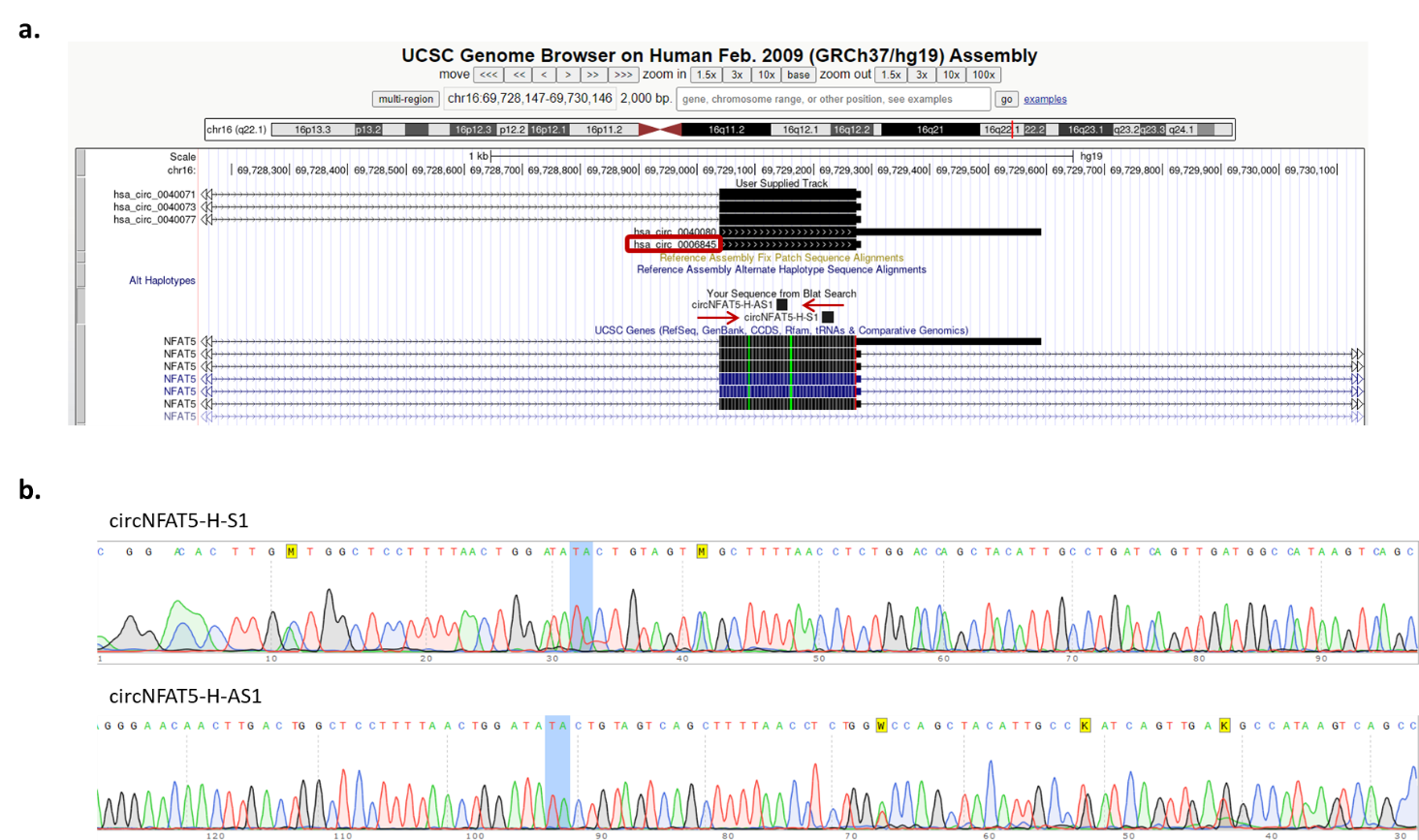


**a.** UCSB representation showing the location of circNFAT5 PCR divergent primers (red arrows), circNFAT5 (hsa_circ_0006845; red square) and linear NFAT5 isoforms. **b.** PCR product of Sanger sequencing using circNFAT5 divergent primers with the junction site highlighted in blue.

Supplemental Figure 5. CircNFAT5 expression levels in 542 patients of the TTM-trial using mRS score


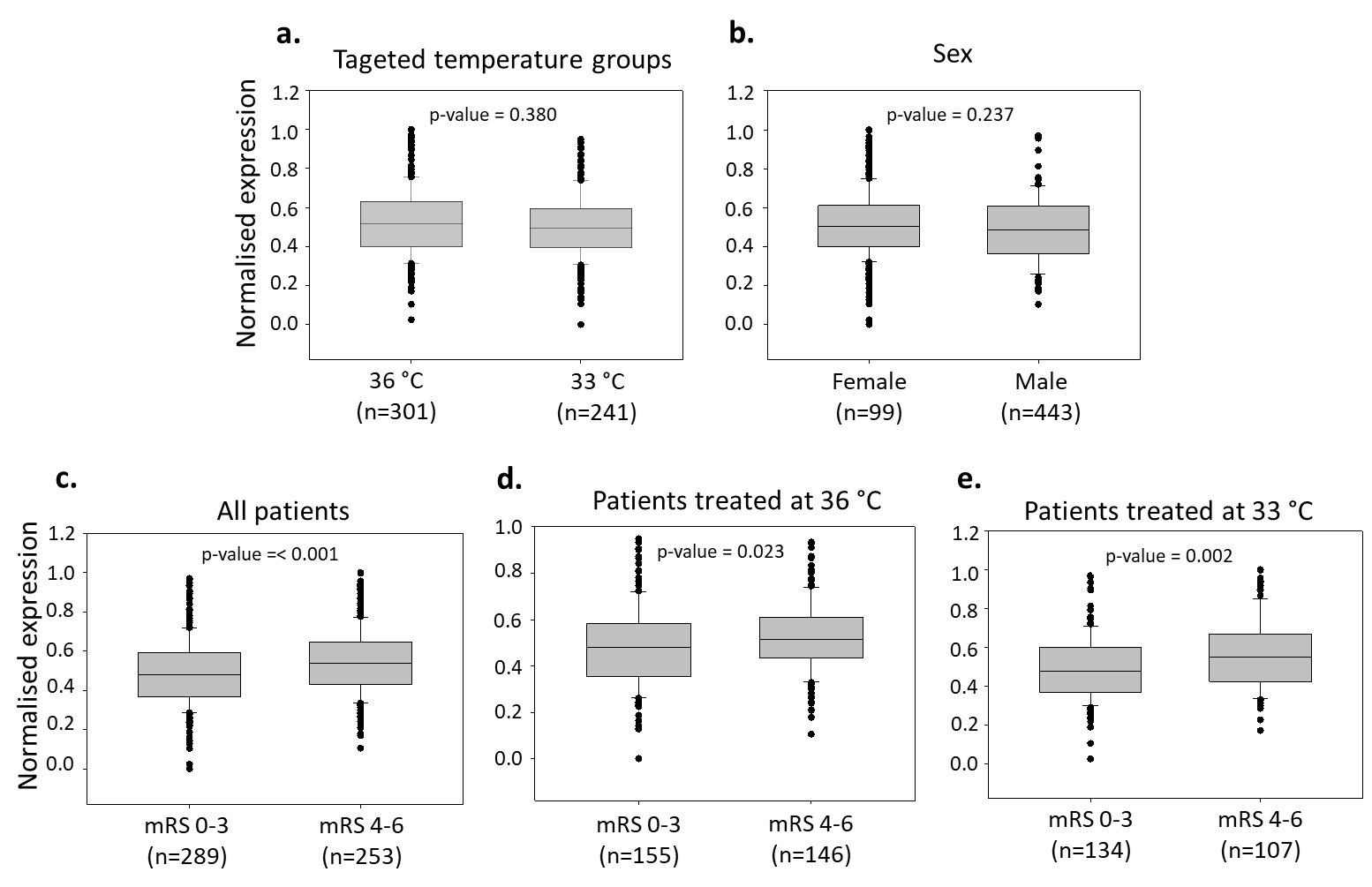


CircNFAT5 levels were compared according to the temperature regimen and regardless of the neurological outcome **(a)**, between females and males **(b),** between good (mRS 0-3) and poor (mRS 3-5) neurological outcome regardless of the temperature regimen **(c),** and separately in patients treated at 36°C or 33°C **(d-e)**. The analyses have been performed as described in figure 2.

Supplemental Figure 6. Logistic regression models to predict neurological outcome in TTM-trial patients.

**
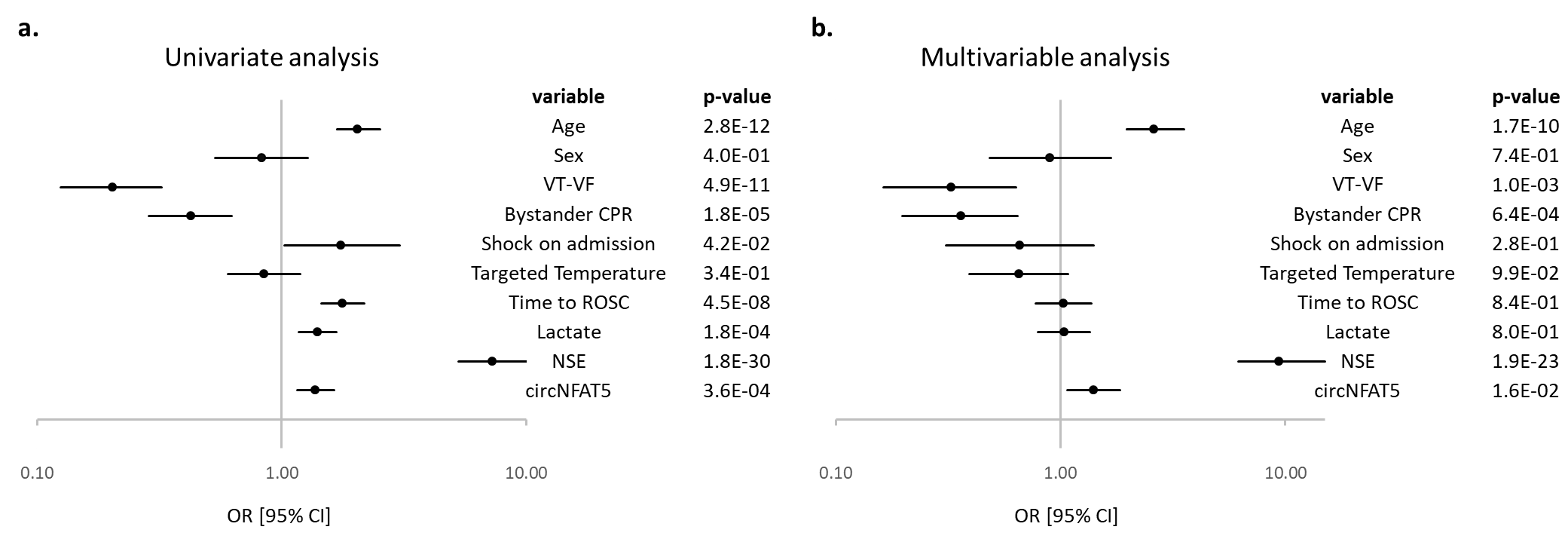
**

Forest plots showing the odds ratio (OR) with ± 95% confidence interval [95% CI] for the prediction of 6-month neurological outcome in TTM-trial patients using mRS score. **a)** Univariate logistic regression analysis. **b)** Multivariable logistic regression analysis.
